# Supplementary material for: Structural basis of actin filament assembly and aging
Source: Nature. 2022 Oct 26;611(7935):374–9. doi: 10.1038/s41586-022-05241-8 (PMC9646518; doi:10.1038/s41586-022-05241-8)
Supplement: Supplementary file 1 — Supplementary Tables 1 and 2 and Fig. 1. [file 41586_2022_5241_MOESM1_ESM.pdf]

nature portfolio

https://doi.org/10.1038/s41586-022-05241-8

Supplementary information

Structural basis of actin filament assembly and aging

In the format provided by the authors and unedited

Supplementary Table 1. Cryo-EM data collection, refinement and validation statistics for Mg^{2+}-F-actin datasets.

**F-actin state**	**Mg^{2+}-ADP-BeF_3^-**<br>EMD-15104<br>PDB 8A2R	**Mg^{2+}-ADP-P_i**<br>EMD-15105<br>PDB 8A2S	**Mg^{2+}-ADP**<br>EMD-15106<br>PDB 8A2T
**Data collection and processing**			
Magnification	130,000	130,000	130,000
Voltage (kV)	300	300	300
Electron exposure (e–/Å^2)	78.9	88.8	76.4
Defocus range (μm)	-0.7 to -2.0	-0.7 to -2.0	-0.7 to -2.0
Pixel size (Å)	0.695	0.695	0.695
Symmetry imposed	C1	C1	C1
Initial particle images (no.)	2,897,679	2,349,979	1,296776
Final particle images (no.)	2,228,553	1,808,554	1,114,051
Map resolution (Å)<br>0.143 FSC threshold	2.17	2.22	2.24
Map resolution range (Å)	2.1 – 3.2	2.2 – 3.2	2.2 – 3.4
Measured helical symmetry			
Helical rise (Å)	27.47±0.05	27.50±0.02	27.50±0.07
Helical twist (°)	166.6±0.2	166.5±0.4	166.7±0.1
**Refinement**			
Initial model used (PDB code)	8A2Z	8A2Z	8A2Z
Model resolution (Å)<br>0.5 FSC threshold	2.20	2.28	2.35
Map sharpening *B* factor (Å^2)	-50	-64	-65
Model composition			
Non-hydrogen atoms	14,990	15,141	15,046
Protein residues	1,830	1,835	1,830
Waters	480	591	556
Ligands	15	15	10
B factors (Å^2)			
Protein	31.53	34.56	38.07
Waters	31.82	23.19	31.98
Ligand	22.89	31.58	31.71
R.m.s. deviations			
Bond lengths (Å)	0.004	0.008	0.003
Bond angles (°)	0.592	0.669	0.549
Validation			
EM-ringer score	5.94	6.45	5.89
MolProbity score	1.17	1.12	1.03
Clashscore	2.75	3.33	2.50
Poor rotamers (%)	0.65	0.64	0.32
Ramachandran plot			
Favored (%)	97.49	98.06	98.05
Allowed (%)	2.51	1.94	1.95
Disallowed (%)	0	0	0

Supplementary Table 2. Cryo-EM data collection, refinement and validation statistics for Ca^{2+}-F-actin datasets.

F-actin state	Ca^{2+}-ADP-BeF_3^-<br>EMD-15107<br>PDB 8A2U	Ca^{2+}-ADP-P_i<br>EMD-15108<br>PDB 8A2Y	Ca^{2+}-ADP<br>EMD-15109<br>PDB 8A2Z
**Data collection and processing**			
Magnification	130,000	130,000	130,000
Voltage (kV)	300	300	300
Electron exposure (e–/Å^2)	84.6	88.8	75.6
Defocus range (μm)	-0.7 to -2.0	-0.7 to -2.0	-0.7 to -2.0
Pixel size (Å)	0.695	0.695	0.695
Symmetry imposed	C1	C1	C1
Initial particle images (no.)	2,246,622	3,031,270	1,873,773
Final particle images (no.)	1,719,432	2,171,987	1,073,455
Map resolution (Å)<br>0.143 FSC threshold	2.21	2.15	2.15
Map resolution range (Å)	2.15 – 3.2	2.1 – 3.1	2.1 – 3.5
Measured helical symmetry			
Helical rise (Å)	27.46±0.05	27.47±0.08	27.47±0.05
Helical twist (°)	166.7±0.1	166.6±0.1	166.5±0.1
**Refinement**			
Initial model used (PDB code)	8A2Z	8A2Z	7AHN
Model resolution (Å)<br>0.5 FSC threshold	2.23	2.16	2.17
Map sharpening *B* factor (Å^2)	-55	-55	-55
Model composition			
Non-hydrogen atoms	15,022	15,078	15,060
Protein residues	1,830	1,835	1,830
Waters	512	528	570
Ligands	15	15	10
B factors (Å^2)			
Protein	24.67	21.12	26.49
Waters	23.66	23.30	28.02
Ligand	16.14	15.86	20.53
R.m.s. deviations			
Bond lengths (Å)	0.003	0.004	0.003
Bond angles (°)	0.542	0.596	0.568
Validation			
EM-ringer score	6.31	6.39	6.56
MolProbity score	1.17	1.02	0.99
Clashscore	2.75	2.39	1.81
Poor rotamers (%)	0.65	0.64	0.00
Ramachandran plot			
Favored (%)	97.49	98.06	97.77
Allowed (%)	2.51	1.94	1.95
Disallowed (%)	0	0	0.28

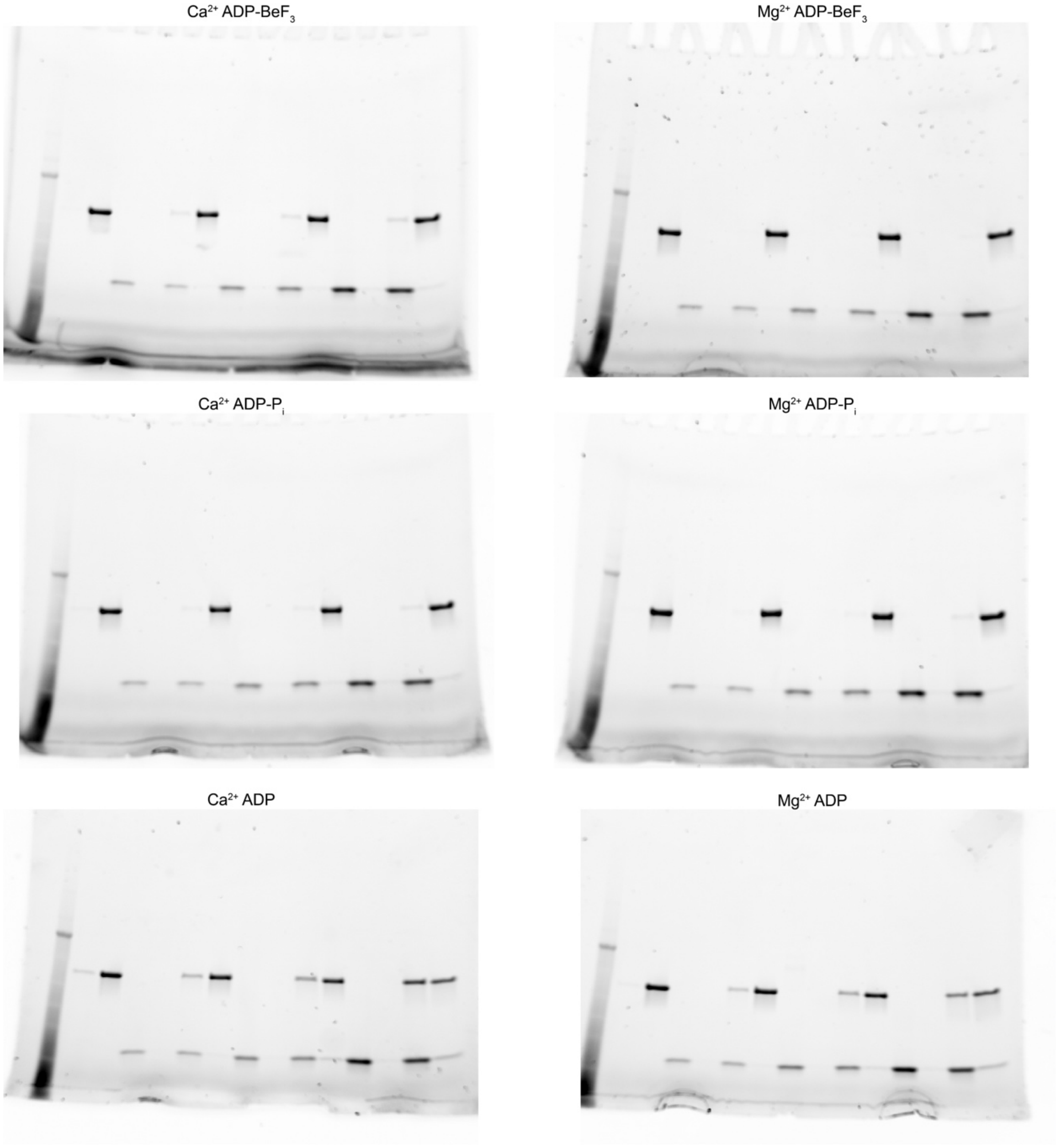

Supplementary Figure 1. Uncropped gel images.
This figure depicts uncropped versions of the gels shown in Extended Data Fig. 10a.
