# Supplementary material for: Structural basis of actin filament assembly and aging
Source: Nature. 2022 Oct 26;611(7935):374–9. doi: 10.1038/s41586-022-05241-8 (PMC9646518; doi:10.1038/s41586-022-05241-8)
Supplement: Supplementary file 2 — Reporting Summary [file 41586_2022_5241_MOESM2_ESM.pdf]

nature portfolio

Corresponding author(s): Stefan Raunser

Last updated by author(s): Aug 4, 2022

Reporting Summary

Nature Portfolio wishes to improve the reproducibility of the work that we publish. This form provides structure for consistency and transparency in reporting. For further information on Nature Portfolio policies, see our Editorial Policies and the Editorial Policy Checklist.

Statistics

For all statistical analyses, confirm that the following items are present in the figure legend, table legend, main text, or Methods section.

n/a	Confirmed	
☐	☒	The exact sample size (n) for each experimental group/condition, given as a discrete number and unit of measurement
☐	☒	A statement on whether measurements were taken from distinct samples or whether the same sample was measured repeatedly
☒	☐	The statistical test(s) used AND whether they are one- or two-sided<br>*Only common tests should be described solely by name; describe more complex techniques in the Methods section.*
☒	☐	A description of all covariates tested
☒	☐	A description of any assumptions or corrections, such as tests of normality and adjustment for multiple comparisons
☐	☒	A full description of the statistical parameters including central tendency (e.g. means) or other basic estimates (e.g. regression coefficient) AND variation (e.g. standard deviation) or associated estimates of uncertainty (e.g. confidence intervals)
☒	☐	For null hypothesis testing, the test statistic (e.g. F, t, r) with confidence intervals, effect sizes, degrees of freedom and P value noted<br>*Give P values as exact values whenever suitable.*
☒	☐	For Bayesian analysis, information on the choice of priors and Markov chain Monte Carlo settings
☒	☐	For hierarchical and complex designs, identification of the appropriate level for tests and full reporting of outcomes
☒	☐	Estimates of effect sizes (e.g. Cohen's d, Pearson's r), indicating how they were calculated

Our web collection on statistics for biologists contains articles on many of the points above.

Software and code

Policy information about availability of computer code

Data collection	Cryo-EM data was collected using the commercially available software EPU version 2.8 (Thermofisher Scientific).
Data analysis	Cryo-EM data collection was monitored and preprocessed on the fly using TranSPHIRE version 1.5.13. The preprocessing steps in TranSPHIRE involved gain and drift correction using UCSF MotionCor2 v1.3.0, CTF estimation with CTFFIND4 v4.1.13, and particle picking using SPHIRE-crYOLO v1.5.8. The data was further processed using helical SPHIRE v1.4 and RELION v3.1.0. Protein model building was performed in COOT v0.8.9.2 and the models were refined using phenix real-space refine v1.20.1-4487. Solvent cavities in protein structures were calculated through the CASTp 3.0 web server. Protein models were validated within the phenix suite v1.20.1-4487. Figures and videos that depict cryo-EM density maps and protein structures were prepared using UCSF ChimeraX v1.3.<br><br>Densitometry of SDS PAGE gel bands in cofilin-severing assays was performed with Image Lab software v6.0.1 (Bio-Rad) and the analysis was done in GrapPad Prism v9 (GraphPad Software).

For manuscripts utilizing custom algorithms or software that are central to the research but not yet described in published literature, software must be made available to editors and reviewers. We strongly encourage code deposition in a community repository (e.g. GitHub). See the Nature Portfolio guidelines for submitting code & software for further information.

Data

Policy information about availability of data

All manuscripts must include a data availability statement. This statement should provide the following information, where applicable:

- Accession codes, unique identifiers, or web links for publicly available datasets
- A description of any restrictions on data availability
- For clinical datasets or third party data, please ensure that the statement adheres to our policy

The cryo-EM maps have been deposited to the Electron Microscopy Data Bank (EMDB) under accession codes (dataset in brackets): EMD-15104 (Mg2+-ADP-BeF3-) [https://www.ebi.ac.uk/emdb/EMD-15104], EMD-15105 (Mg2+-ADP-Pi) [https://www.ebi.ac.uk/emdb/EMD-15105], EMD-15106 (Mg2+-ADP) [https://www.ebi.ac.uk/emdb/EMD-15106], EMD-15107 (Ca2+-ADP-BeF3-) [https://www.ebi.ac.uk/emdb/EMD-15107], EMD-15108 (Ca2+-ADP-Pi) [https://www.ebi.ac.uk/emdb/EMD-15108] and EMD-15109 (Ca2+-ADP) [https://www.ebi.ac.uk/emdb/EMD-15109]. These depositions include sharpened maps, unfiltered half-maps and the refinement masks. For the Mg2+-ADP-BeF3- F-actin submission, all density maps and masks regarding the separation of open/closed D-loop conformations are provided. The atomic coordinates of the protein structures have been submitted to the Protein Data Bank (PDB) under accession codes (dataset in brackets): 8A2R (Mg2+-ADP-BeF3-) [https://doi.org/10.2210/pdb8A2R/pdb], 8A2S (Mg2+-ADP-Pi) [https://doi.org/10.2210/pdb8A2S/pdb], 8A2T (Mg2+-ADP) [https://doi.org/10.2210/pdb8A2T/pdb], 8A2U (Ca2+-ADP-BeF3-) [https://doi.org/10.2210/pdb8A2U/pdb], 8A2Y (Ca2+-ADP-Pi) [https://doi.org/10.2210/pdb8A2Y/pdb] and 8A2Z (Ca2+-ADP) [https://doi.org/10.2210/pdb8A2Z/pdb]. We used the following previously published structures for modeling and comparisons: 7AHN [https://doi.org/10.2210/pdb7AHN/pdb], 2V52 [https://doi.org/10.2210/pdb2V52/pdb], 6RSW [https://doi.org/10.2210/pdb6RSW/pdb], 1QZ5 [https://doi.org/10.2210/pdb1QZ5/pdb], 1J6Z [https://doi.org/10.2210/pdb1J6Z/pdb]. EMD-11787 [https://www.ebi.ac.uk/emdb/EMD-11787] was used as initial model for the first 3D refinement. Source data are provided with this paper.

Field-specific reporting

Please select the one below that is the best fit for your research. If you are not sure, read the appropriate sections before making your selection.

☒ Life sciences ☐ Behavioural & social sciences ☐ Ecological, evolutionary & environmental sciences

For a reference copy of the document with all sections, see nature.com/documents/nr-reporting-summary-flat.pdf

Life sciences study design

All studies must disclose on these points even when the disclosure is negative.

Sample size	Sample sizes for the six cryo-EM datasets presented in this study: For the Mg-ADP-BeF3- F-actin dataset, 10,822 micrographs were collected. 2,897,679 total particles were picked and 2,228,553 particles were used for the final reconstruction. For the Mg-ADP-Pi F-actin dataset, 9,658 micrographs were collected. 2,349,979 total particles were picked and 1,808,554 particles were used for the final reconstruction. For the Mg-ADP F-actin dataset, 9,842 micrographs were collected. 1,296,776 total particles were picked and 1,114,051 particles were used for the final reconstruction. For the Ca-ADP-BeF3- F-actin dataset, 10,705 micrographs were collected. 2,246,622 total particles were picked and 1,719,432 particles were used for the final reconstruction. For the Ca-ADP-Pi F-actin dataset, 10,156 micrographs were collected. 3,031,270 total particles were picked and 2,171,987 particles were used for the final reconstruction. For the Ca-ADP F-actin dataset, 10,733 micrographs were collected. 1,873,773 total particles were picked and 1,073,455 particles were used for the final reconstruction. These sample sizes of ~10,000 micrographs are common in the cryo-EM field for obtaining high-resolution protein structures, see for example Belyy et al. Nat. Commun. (2021): https://doi.org/10.1038/s41467-021-26889-2.<br><br>The cofilin-severing assays were performed as three independent experiments. The sample size of n=3 is common for in vitro assays with purified proteins, see for example Belyy et al. Plos Biol. (2020): https://doi.org/10.1371/journal.pbio.3000925
Data exclusions	During the cryo-EM image processing, particles that represented false picks or particles that did not contribute high-resolution information to the reconstructions were discarded through 2D and 3D classification procedures. This process, which is required to obtain high-resolution reconstructions, is a standard procedure in cryo-EM image processing.
Replication	All cryo-EM datasets were collected in one session per F-actin functional state and were not repeated. It is unattainable from a time and cost perspective to repeat cryo-EM data collection and processing on the exact same sample.<br><br>The cofilin-severing assays were performed in triplicate. They were performed as independent experiments, with new protein aliquots from the same purification batch.<br>All attempts at replication were successful.
Randomization	For the 3D refinement of cryo-EM structures, particles were randomly split into two half sets. For all other experiments, randomization was not required because all data were used in the analysis. Covariates were not controlled.
Blinding	This study does not involve any experiments where blinding would be applicable.

Behavioural & social sciences study design

All studies must disclose on these points even when the disclosure is negative.

Study description	*Briefly describe the study type including whether data are quantitative, qualitative, or mixed-methods (e.g. qualitative cross-sectional, quantitative experimental, mixed-methods case study).*
Research sample	*State the research sample (e.g. Harvard university undergraduates, villagers in rural India) and provide relevant demographic information (e.g. age, sex) and indicate whether the sample is representative. Provide a rationale for the study sample chosen. For studies involving existing datasets, please describe the dataset and source.*
Sampling strategy	*Describe the sampling procedure (e.g. random, snowball, stratified, convenience). Describe the statistical methods that were used to predetermine sample size OR if no sample-size calculation was performed, describe how sample sizes were chosen and provide a rationale for why these sample sizes are sufficient. For qualitative data, please indicate whether data saturation was considered, and what criteria were used to decide that no further sampling was needed.*
Data collection	*Provide details about the data collection procedure, including the instruments or devices used to record the data (e.g. pen and paper, computer, eye tracker, video or audio equipment) whether anyone was present besides the participant(s) and the researcher, and whether the researcher was blind to experimental condition and/or the study hypothesis during data collection.*
Timing	*Indicate the start and stop dates of data collection. If there is a gap between collection periods, state the dates for each sample cohort.*
Data exclusions	*If no data were excluded from the analyses, state so OR if data were excluded, provide the exact number of exclusions and the rationale behind them, indicating whether exclusion criteria were pre-established.*
Non-participation	*State how many participants dropped out/declined participation and the reason(s) given OR provide response rate OR state that no participants dropped out/declined participation.*
Randomization	*If participants were not allocated into experimental groups, state so OR describe how participants were allocated to groups, and if allocation was not random, describe how covariates were controlled.*

Ecological, evolutionary & environmental sciences study design

All studies must disclose on these points even when the disclosure is negative.

Study description	*Briefly describe the study. For quantitative data include treatment factors and interactions, design structure (e.g. factorial, nested, hierarchical), nature and number of experimental units and replicates.*
Research sample	*Describe the research sample (e.g. a group of tagged Passer domesticus, all Stenocereus thurberi within Organ Pipe Cactus National Monument), and provide a rationale for the sample choice. When relevant, describe the organism taxa, source, sex, age range and any manipulations. State what population the sample is meant to represent when applicable. For studies involving existing datasets, describe the data and its source.*
Sampling strategy	*Note the sampling procedure. Describe the statistical methods that were used to predetermine sample size OR if no sample-size calculation was performed, describe how sample sizes were chosen and provide a rationale for why these sample sizes are sufficient.*
Data collection	*Describe the data collection procedure, including who recorded the data and how.*
Timing and spatial scale	*Indicate the start and stop dates of data collection, noting the frequency and periodicity of sampling and providing a rationale for these choices. If there is a gap between collection periods, state the dates for each sample cohort. Specify the spatial scale from which the data are taken*
Data exclusions	*If no data were excluded from the analyses, state so OR if data were excluded, describe the exclusions and the rationale behind them, indicating whether exclusion criteria were pre-established.*
Reproducibility	*Describe the measures taken to verify the reproducibility of experimental findings. For each experiment, note whether any attempts to repeat the experiment failed OR state that all attempts to repeat the experiment were successful.*
Randomization	*Describe how samples/organisms/participants were allocated into groups. If allocation was not random, describe how covariates were controlled. If this is not relevant to your study, explain why.*
Blinding	*Describe the extent of blinding used during data acquisition and analysis. If blinding was not possible, describe why OR explain why blinding was not relevant to your study.*

Did the study involve field work? ☐ Yes ☒ No

Reporting for specific materials, systems and methods

We require information from authors about some types of materials, experimental systems and methods used in many studies. Here, indicate whether each material, system or method listed is relevant to your study. If you are not sure if a list item applies to your research, read the appropriate section before selecting a response.

Materials & experimental systems

n/a	Involved in the study
☒	☐ Antibodies
☒	☐ Eukaryotic cell lines
☒	☐ Palaeontology and archaeology
☒	☐ Animals and other organisms
☒	☐ Human research participants
☒	☐ Clinical data
☒	☐ Dual use research of concern

Methods

n/a	Involved in the study
☒	☐ ChIP-seq
☒	☐ Flow cytometry
☒	☐ MRI-based neuroimaging
